# Acetabular Fracture Following Total Hip Replacement in a Patient With Neglected Developmental Dysplasia of the Hip: A Case Report

**DOI:** 10.1155/cro/1254882

**Published:** 2026-05-24

**Authors:** Marah M. Alomari, Rahaf E. Farah, Rinad B. Shahen, Rou′a E.Farah, Mutasem Iqnaibi

**Affiliations:** ^1^ Palestine Polytechnic University College of Medicine and Health Science, Hebron, State of Palestine; ^2^ Operation and Anesthesia Department, Alia Hospital, Hebron, State of Palestine

**Keywords:** case report, neglected DDH, periprosthatic fracture, spontaneous, THA

## Abstract

Neglected DDH can cause degenerative changes to the hip joint that could result in subsequent hip pain and disability. The best treatment for these patients is total hip arthroplasty (THA) with artificial joint prosthesis implantation. Acetabular fractures in dysplastic hips with THA are uniquely complex, being more unstable, difficult to assess radiographically due to implant interference, and often atypical in pattern. Management is highly challenging, as treatment decisions must balance surgical risks with prosthetic stability and patient factors, making the successful conservative recovery in this case both rare and notable. This case report presents a case of a 42‐year‐old female patient with neglected DDH who underwent bilateral total hip replacement complicated by a spontaneous acetabular fracture. Radiographs revealed a nondisplaced, spontaneous acetabular fracture in the superior region. Based on the timing and implant stability, the injury was classified as a Pascarella Type 2A. Due to the clinical context and surgical complexity in dysplastic hips with prior THA, the patient was treated conservatively consisting of protected weight‐bearing, rehabilitation, and close follow‐up, with full improvement.

## 1. Introduction

Developmental dysplasia of the hip (DDH) is the most common musculoskeletal disorder in infants, with an incidence ranging from 0.5% to 1.5% [1]. In asymptomatic patients, DDH may remain undetected during infancy and childhood, leading to delayed diagnosis [2]. Neglected DDH can result in early hip osteoarthritis, limb length discrepancies, limping, and significant disability [3]. Total hip arthroplasty (THA) with artificial joint prosthesis implantation is the optimal treatment for such patients [4].

However, THA in adults with DDH can be associated with complications, including periprosthetic fractures, which occur with an incidence of 0.4%–3.5% [5]. While periprosthetic fractures of the femur are relatively common (7.8%), acetabular fractures are significantly less frequent (0.8%) but can be catastrophic [6]. Repairing acetabular fractures in the presence of hip arthroplasty poses complex challenges for orthopedic surgeons [7].

In this paper, we present a case of a spontaneous acetabular fracture following THA in a patient with neglected DDH. This case was managed conservatively, resulting in full recovery and highlights the potential for successful nonsurgical management of such complications.

## 2. Case Presentation

A 42‐year‐old female presented with a 3‐month history of severe right‐sided hip and subgluteal pain, persistent and aggravated by movement, with associated limping and restricted range of motion. The pain was unresponsive to NSAIDs and opioids. Her past medical history included hypertension and polycystic ovary syndrome, for which she was receiving amlodipine 5 mg and bisoprolol 5 mg daily.

At the age of 29, she was diagnosed with neglected DDH after experiencing progressive left‐sided hip pain. She was initially managed conservatively with analgesics but later developed worsening symptoms following her third pregnancy and a minor fall.

At the age of 41, due to severe left hip osteoarthritis, she underwent left total hip replacement (THR) with cemented components. The postoperative course was uneventful, with a stable prosthesis on follow‐up radiographs. Functionally, she regained independent ambulation, requiring only intermittent NSAIDs for mild contralateral (right) hip pain.

**Figure 1:**
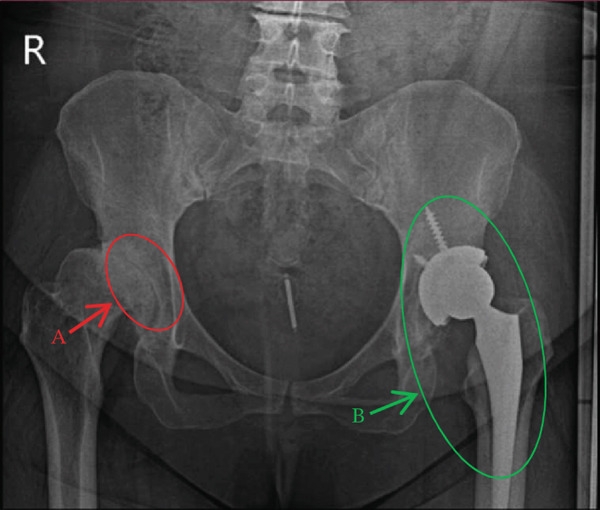
Pseudoarticulation with advanced degenerative changes secondary to dislocation in the right hip joint (A). The left hip joint shows hip replacement with screws (B).

**Figure 2:**
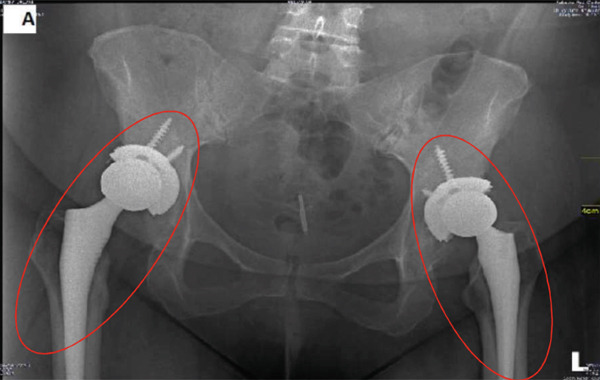
Bilateral total hip replacement without complication.

One year later (age 42), she developed progressive right hip pain. Examination revealed right hip tenderness and restricted movement. Radiographs demonstrated pseudoarticulation with advanced degenerative changes secondary to dislocation and osteoarthritis of the right hip. The left hip joint shows hip replacement with screws (Figure 1), and she subsequently underwent right THR. The immediate postoperative course was initially satisfactory (Figure 2).

**Figure 3:**
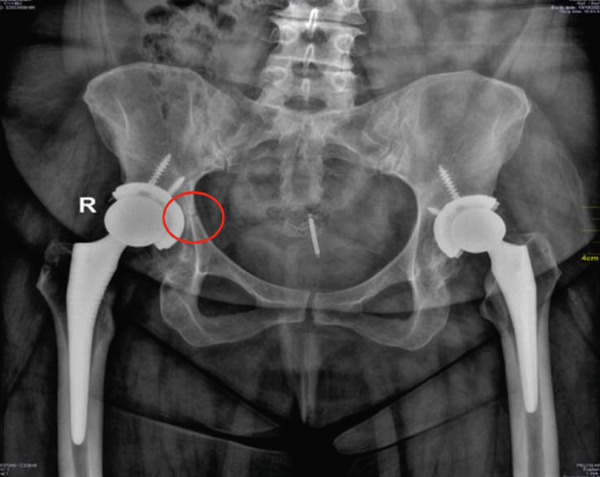
Right acetabulum: Evidence of acetabular periprosthetic fracture adjacent to the right hip arthroplasty cup, most likely involving the superior acetabular region. Prosthetic components appear stable.

Two weeks postoperatively, however, the patient experienced recurrent severe right hip pain refractory to NSAIDs and opioids. The visual analog scale was 10/10 [8]. On follow‐up, physical examination showed tenderness around the surgical site and significant restriction of hip motion, and the adductor muscle shows severe tenderness. Radiographs (Figure 3) revealed an acetabular fracture with loosening of the right prosthetic cup. She denied any history of trauma or fall during this period.

At the time of reporting, the left THR remained stable and functional, whereas the right THR was complicated by early prosthetic loosening and acetabular fracture, leading to severe pain and significant limitation of mobility.

Active management was initiated with an interventional block of the right lateral femoral cutaneous nerve and the right obturator nerve. Done with lidocaine and kenalog 20 mg. NSAID, tramadol, and codeine were held. Started gabapentin 200 mg daily, baclofen tablets 10 mg/12 h daily, regular acetaminophen 1000 mg/8 h, acetylsalicylic acid 100 mg/12 h for 10 days, levofloxacin 500 mg/12 h for 1 week (due to an incision site wound infection), calcium citrate 600 mg daily, vitamin K2 120 mcg once daily, and vitamin D3 5000 IU once daily. The patient was partial weight‐bearing with the assistance of a walker.

As a result of the management plan, the pain directly started to subside, and the visual analog scale became 3/10.[8].

After 2 weeks, the patient came for follow‐up with an improvement of visual analog scale 1/10 [8]. And all through her last period, she started to walk with a weight‐bearing walker. At this time, only gabapentin was increased to 300 mg daily. Pelvic x‐ray (Figure 4a) shows healing and callous formation.

Figure 4(a, b) Right acetabulum: Periprosthetic fracture line is seen adjacent to the acetabular cup, with surrounding callus formation consistent with the healing phase. No obvious displacement. Acetabular screws and cup remain in stable alignment without migration.

**Figure:**
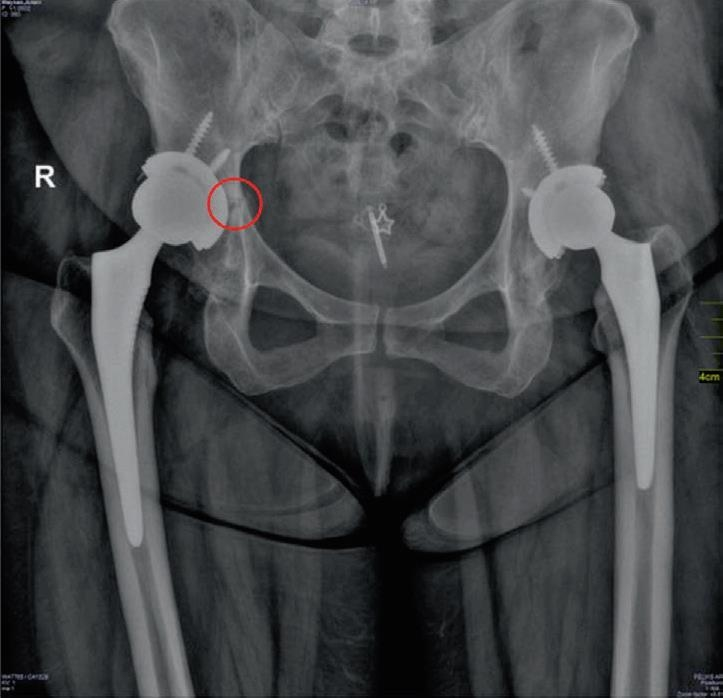
(a)

**Figure:**
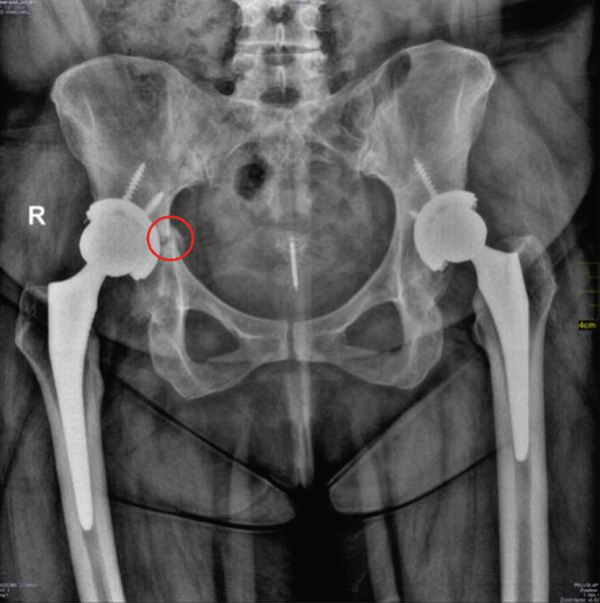
(b)

After 2 weeks, her visual analog scale was 0/10, and she was walking independently [8]. The management plan kept the same as analgesics and food supplement bills, with advice for her to keep a weight‐bearing walker.

In the last visit, the final result of the management plan and lifestyle modification she was walking independently and without pain. Control x‐ray shows complete healing of the fracture (Figure 4b).

The timeline event is demonstrated in Table 1.

**Table 1:** Timeline table.

Time point	Clinical events
Age 29	First diagnosis of neglected DDH after progressive left‐sided hip pain; managed conservatively.
Following years	Progressive symptoms after pregnancy and minor fall.
Age 41	Left THR performed for severe OA. Post‐op recovery uneventful; stable prosthesis.
Age 42	Progressive right hip pain; radiographs show pseudo‐articulation and degenerative changes.
Right THR performed	Immediate post‐op course initially satisfactory.
2 weeks postright THR	Sudden severe right hip pain (VAS 10/10); exam: severe tenderness, restricted ROM.
Radiographs obtained	Acetabular periprosthetic fracture with concern for early cup loosening. No trauma reported.
Initial management	Nerve blocks (LFCN + obturator), analgesics, gabapentin, baclofen, partial weight‐bearing with walker.
2 weeks after intervention	Pain improves to VAS 3/10; patient able to ambulate with support. Radiograph shows early callus formation.
4 weeks after intervention	Pain VAS 1/10; callus progression on imaging; walking with walker.
6 weeks after intervention	VAS 0/10, independent walking; final X‐ray shows complete fracture healing.

## 3. Discussion

DDH is a complex condition that can lead to improper hip joint development and significant complications in adulthood, such as hip pain, limping, and mechanical symptoms like catching or popping sensations [9]. Diagnosis can be delayed due to the gradual onset of symptoms, resulting in secondary osteoarthritis in middle age if left untreated. The classification systems by Hartofilakidis and Crowe are commonly used to characterize DDH. Hartofilakidis classifies DDH into three grades based on the femoral head′s coverage by the acetabulum, while Crowe classifies it into four grades based on the migration of the femoral head–neck junction from the interteardrop line. Diagnosis typically involves radiographic analysis, which assesses acetabular depth and femoral head displacement, and may also include MRI to evaluate cartilage and labrum damage. Treatment for hip dysplasia varies based on severity. Mild cases might improve with weight loss, exercise, and physical therapy, whereas severe cases often require surgical intervention, such as THR. Periprosthetic fractures are a known complication of THA, with the incidence rising due to the increased number of arthroplasty surgeries and the higher expectations of older patients. Diagnosis of periprosthetic fractures typically involves plain radiographs, and treatment depends on the fracture′s location, implant stability, and bone stock. Nonsurgical management is possible for certain fractures, while others require surgical intervention.

In the presented case, a 42‐year‐old female with neglected DDH experienced a spontaneous acetabular fracture post‐THA. This is a rare but serious complication, given the lower incidence of acetabular fractures compared to femoral fractures [7]. The spontaneous nature of the fracture, with no reported history of trauma or fall, suggests it was likely a stress fracture resulting from underlying factors. Causes and consequences: These factors include compromised or dysplastic bone stock characteristic of neglected DDH, atypical stress distribution on the bone following THA, and potentially the initial radiographic finding of early prosthetic loosening of the cup. The consequences were recurrent severe right hip pain refractory to NSAIDs and opioids (Visual Analog Scale 10/10) [8] and significant restriction of hip motion. Acetabular fractures in dysplastic hips with THA are uniquely complex, being more unstable, difficult to assess radiographically due to implant interference, and often atypical in pattern [4].

The reason to consider loosening of the acetabular cup stemmed from the initial radiographic evidence (Figure 3) which revealed the fracture accompanied by a concern for loosening of the right prosthetic cup. This loosening may be a cause or a consequence, as the fracture itself, especially in the superior acetabular region (Figure 3), could compromise the structural support of the cup. However, follow‐up control x‐rays (Figure 4a,b) later showed the fracture healing and callus formation, with the acetabular screws and cup remaining in stable alignment without migration. This demonstrated stability, despite initial concerns, was crucial in supporting the conservative approach.

The patient′s fracture was managed conservatively, with pain management and physical therapy, leading to successful healing and full recovery without additional surgical intervention. The rationale behind the chosen treatment approach was the need to balance the high surgical risks associated with revision surgery in a complex dysplastic hip against the potential for healing, especially given the lack of severe fracture displacement and the aggressive initial pain management strategy. Due to the clinical context and surgical complexity in dysplastic hips with prior THA, the patient was treated conservatively [7]. The successful conservative recovery in this case is both rare and notable [5]. The aggressive nonsurgical plan included protected weight‐bearing, rehabilitation, and an interventional block of the right lateral femoral cutaneous and obturator nerves, alongside oral medications (gabapentin, baclofen, acetaminophen, etc.) and supplements to support bone health. The pain subsided rapidly following this plan, with the visual analog scale dropping from 10/10 to 3/10 [8].

This case underscores the importance of early recognition and appropriate management of post‐THA complications. Conservative management, including interventional pain management and medication, can be effective in treating spontaneous acetabular fractures, particularly in patients with complex histories like neglected DDH. It also highlights the need for careful monitoring and follow‐up in patients undergoing THA to promptly identify and address potential complications. The successful outcome in this case suggests that conservative management should be considered a viable option in similar cases, potentially avoiding the risks and challenges associated with additional surgery. Further studies and case reports are necessary to establish standardized guidelines for the management of such rare complications.

## 4. Conclusion

Spontaneous acetabular fractures post‐THA in neglected DDH patients are rare but serious. This case validates that conservative management is a viable and highly successful alternative to high‐risk surgical revision when acetabular cup stability is confirmed via imaging (the critical indication). The benefit of this approach is primarily avoiding the high technical and medical risks associated with operating on compromised, dysplastic bone stock. Success was achieved through aggressive nonoperative methods, including interventional pain management and protected weight‐bearing, highlighting the crucial need for early diagnosis and rigorous monitoring in these complex cases.

## Funding

The authors declare that writing and publishing this manuscript was not funded by any organization.

## Ethics Statement

This was a case report approved by Palestine Polytechnic University. Informed consent was obtained from the patient for the publication of this case report and any accompanying images. The patient′s anonymity has been preserved throughout the manuscript.

## Conflicts of Interest

The authors declare no conflicts of interest.

## Data Availability

The data that support the findings of this study are available from the corresponding author upon reasonable request.
